# Case Report: Durable remission of intraocular mantle cell lymphoma after CD19 CAR-T therapy

**DOI:** 10.3389/fimmu.2026.1786040

**Published:** 2026-03-27

**Authors:** Álvaro Bienert-García, Carolina Arruabarrena, Mónica Asencio-Duran, Marta Morado Arias, Tycho Baumann, Antonia Rodriguez Izquierdo, José María Sánchez-Pina, Nieves López-Muñoz, Daniel Gil-Alós, Adolfo Jesús Saez-Marín, María Calbacho, Joaquín Martínez-López, Ana Jiménez-Ubieto

**Affiliations:** 1 Servicio Hematología, Hospital Universitario 12 de Octubre, imas12, Universidad Complutense, Spanish National Cancer Research Center (CNIO), Madrid, Spain; 2 Servicio Oftalmología, Hospital Universitario Príncipe de Asturias, Universidad de Alcalá de Henares, Madrid, Spain; 3 Servicio Oftalmología, Hospital Universitario La Paz, Madrid, Spain; 4 Servicio Hematología, Hospital Universitario La Paz, Madrid, Spain

**Keywords:** brexucabtageneautoleucel, CAR-T, flow cytometry, intraocular involvement, mantle cell lymphoma, vitrectomy

## Abstract

Mantle cell lymphoma (MCL) is an aggressive B-cell neoplasm with poor prognosis in refractory relapse. Intraocular (vitreoretinal) involvement is extremely rare and represents a diagnostic and therapeutic challenge due to the immune-privileged status of the eye and the risk of central nervous system (CNS) dissemination. Ocular relapse in mantle cell lymphoma has rarely been described, and evidence on the use of CAR-T therapy in this setting is lacking. We report the case of a 61-year-old man with stage IV-B MCL diagnosed in 2018, who experienced multiple relapses and developed intraocular infiltration confirmed by flow cytometric immunophenotyping of vitreous fluid in 2024. After bridging therapy with intravitreal methotrexate, the patient received CD19-directed CAR-T therapy with brexucabtagene autoleucel (Brexucel) as fifth-line treatment, achieving sustained complete metabolic remission with no ocular or systemic relapse after 19 months of follow-up. This case highlights the diagnostic value of flow cytometry in intraocular lymphomas and supports the expanding role of CAR-T therapy in refractory MCL with atypical sanctuary site involvement.

## 1 Introduction

Intraocular lymphomas are a rare entity, with an estimated incidence of 0.47 cases per 1,000,000 persons per year, accounting for <1% of all non-Hodgkin lymphomas and approximately 15% of primary CNS lymphomas (1). They are most commonly diffuse large B-cell lymphoma (DLBCL), presenting as primary vitreoretinal lymphoma or secondary ocular involvement from systemic disease (2). The eye is an immune-privileged site, limiting immune cell trafficking and drug delivery, which complicates diagnosis and management (3). Mantle cell lymphoma (MCL), accounting for 5–7% of non-Hodgkin lymphomas, is driven by t(11;14)(q13;q32) and cyclin-D1 overexpression (4). Intraocular involvement by MCL is exceptionally uncommon, with fewer than 10 cases reported in the literature to date.

Despite intensive frontline therapy, including cytarabine-based chemoimmunotherapy and autologous stem-cell transplantation, most patients relapse. Covalent BTK inhibitors are the standard salvage therapy in first relapse, and emerging treatments such as CD19 CAR-T cells have demonstrated substantial activity even in immune-privileged sites (5–7). Diagnosing intraocular lymphoma is challenging; cytology alone lacks sensitivity, whereas flow cytometry and molecular clonality assays improve detection (8). Here, we report a rare case of intraocular relapse of MCL successfully treated with CD19-directed CAR-T cells.

## 2 Case presentation

**Figure 1:**
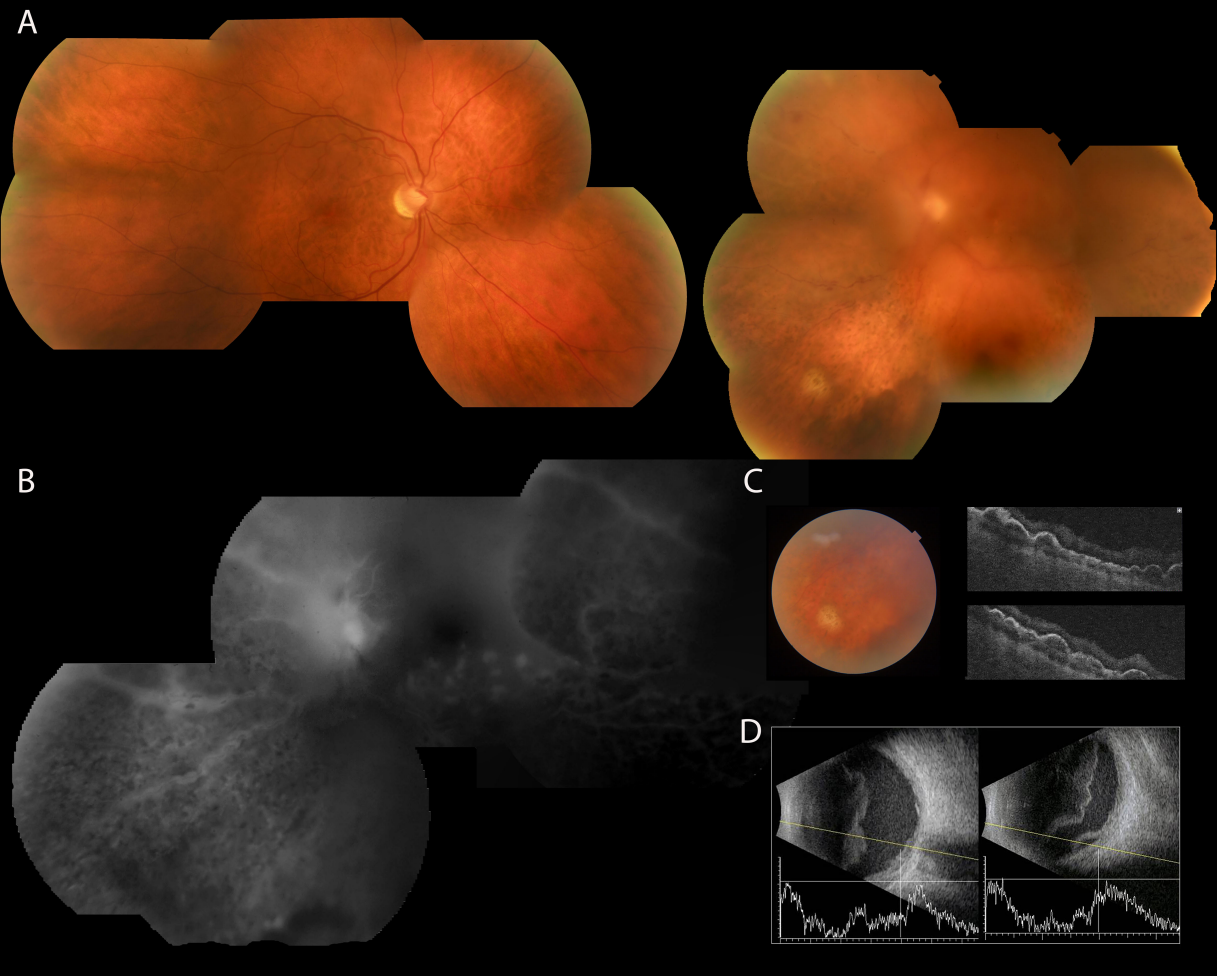
**(A)** A retinographic composition of both eyes reveals an apparently normal fundus in the RE. In contrast, the LE shows vitreous cloudiness due to vitreous hemorrhage, which obscures the details of the posterior pole. However, a flame-shaped hemorrhage is visible at the superior pole of the optic nerve near the superior temporal arcade. Periphlebitis and peripheral ischemia are more pronounced in the nasal quadrants. Notably, there is pigment redistribution in the equator and periphery, with a leopard pattern, along with a rounded yellowish choroidal mass in the inferior nasal quadrant. **(B)** Detail of fluorescein angiography of the left nasal fundus showing dye leakage from the optic nerve and vessels of the inferior temporal arcade toward the posterior pole. Patchy hypofluorescent images in the equator and periphery are consistent with pigment redistribution. Perivenous leakage also occurs in the vessels of the inferior nasal quadrant, as well as areas of ischemia in the inferior nasal quadrants. **(C)** OCT scan of the choroidal mass area shows retinal pigment epithelium undulations. **(D)** B-scan ocular ultrasonography Left: diffuse vitreous hyperreflectivity consistent with vitreous detachment and hemorrhage, and inferior choroidal-retinal thickening Right: detail of the inferior nasal quadrant showing partial retinal detachment.

A 61-year-old man with no personal medical history was diagnosed in 2018 with stage IV-B MCL involving gastric and bone marrow sites (MIPI 6.5, intermediate-risk). Immunohistochemistry showed wild-type p53 expression, and NGS confirmed the absence of TP53 mutations. Histopathology revealed a blastoid variant with Ki-67 of 70%. He received first-line R-VCAP ×3 (rituximab, bortezomib, cyclophosphamide, doxorubicin and prednisone) followed by R-ESHAP (rituximab, etoposide, methylprednisolone, cytarabine and cisplatin), achieving a complete metabolic response (CR) with a Deauville score of 2 according to the Lugano criteria. Consolidation with high-dose cytarabine and BEAM-conditioned (carmustine, etoposide, cytarabine, and melphalan) ASCT (Autologous Stem Cell Transplantation) was performed. Central nervous system (CNS) prophylaxis was not administered. Rituximab maintenance was given for 2 years. In March 2021 he developed a first relapse with extranodal involvement, muscular and colonic, with a high risk (MIPI prognostic index 7.3). After two cycles of HyperCVAD-R (hyperfractionated cyclophosphamide, vincristine, doxorubicin, dexamethasone plus rituximab) alternating with MTX-AraC-R (high-dose methotrexate, cytarabine and rituximab), he achieved a CR. However, because he developed persistent and clinically significant pancytopenia, he was deemed ineligible for allogeneic stem-cell transplantation, which had initially been considered as consolidation therapy. For this reason, ibrutinib 560 mg/day was initiated as maintenance treatment, despite the CR to chemotherapy, in order to provide ongoing disease control in the absence of the planned allogeneic transplant. A second relapse occurred in June 2022 with nodal, muscular and penile lesions. This relapse corresponded to a high-risk (MIPI score 9.6). The biopsy showed CD20-negative, blastoid morphology with wild-type p53 and c-MYC (assessed by immunohistochemistry). He was enrolled in the topMIND trial (NCT2020-005591-35) evaluating tafasitamab + parsaclisib (March 2022). He initially achieved metabolic CR, though later a heterogeneous pattern suggested possible progression. Treatment was discontinued due to trial closure, suspicion of early relapse, and acute hepatitis. Autoimmune etiology was later excluded after HEV infection was identified, resolving with ribavirin. In June 2023, the patient presented with new-onset photopsia and floaters in the left eye (LE), initially consistent with symptomatic posterior vitreous detachment, which subsequently progressed to worsening visual acuity and vitreous hemorrhage. Best corrected visual acuity (BCVA) was light perception in the LE before vitrectomy, while the right eye was 20/20 (Snellen). Fundus photography of the LE (**Figure 1A**) showed vitreous haze, retinal ischemia, periphlebitis and a choroidal mass, while the right eye (RE) examination was unremarkable. Fluorescein angiography (**Figure 1B**) demonstrated areas of vascular leakage corresponding to the periphlebitis and ischemic changes. Optical coherence tomography (OCT) of the choroidal mass area (**Figure 1C**) showed undulations of the retinal pigment epithelium, suggestive of choroidal infiltration. B-scan ultrasonography confirmed vitreous hemorrhage and choroidal thickening (**Figure 1D**).

Given the strong suspicion of intraocular lymphoma, an extensive workup was performed to exclude infectious and autoimmune etiologies. PET-CT showed no evidence of systemic disease, and a bone marrow biopsy confirmed the absence of lymphomatous infiltration. CNS staging included brain MRI and cerebrospinal fluid (CSF) examination; both cytology and flow cytometry were negative, ruling out concurrent CNS relapse.

**Figure 2:**
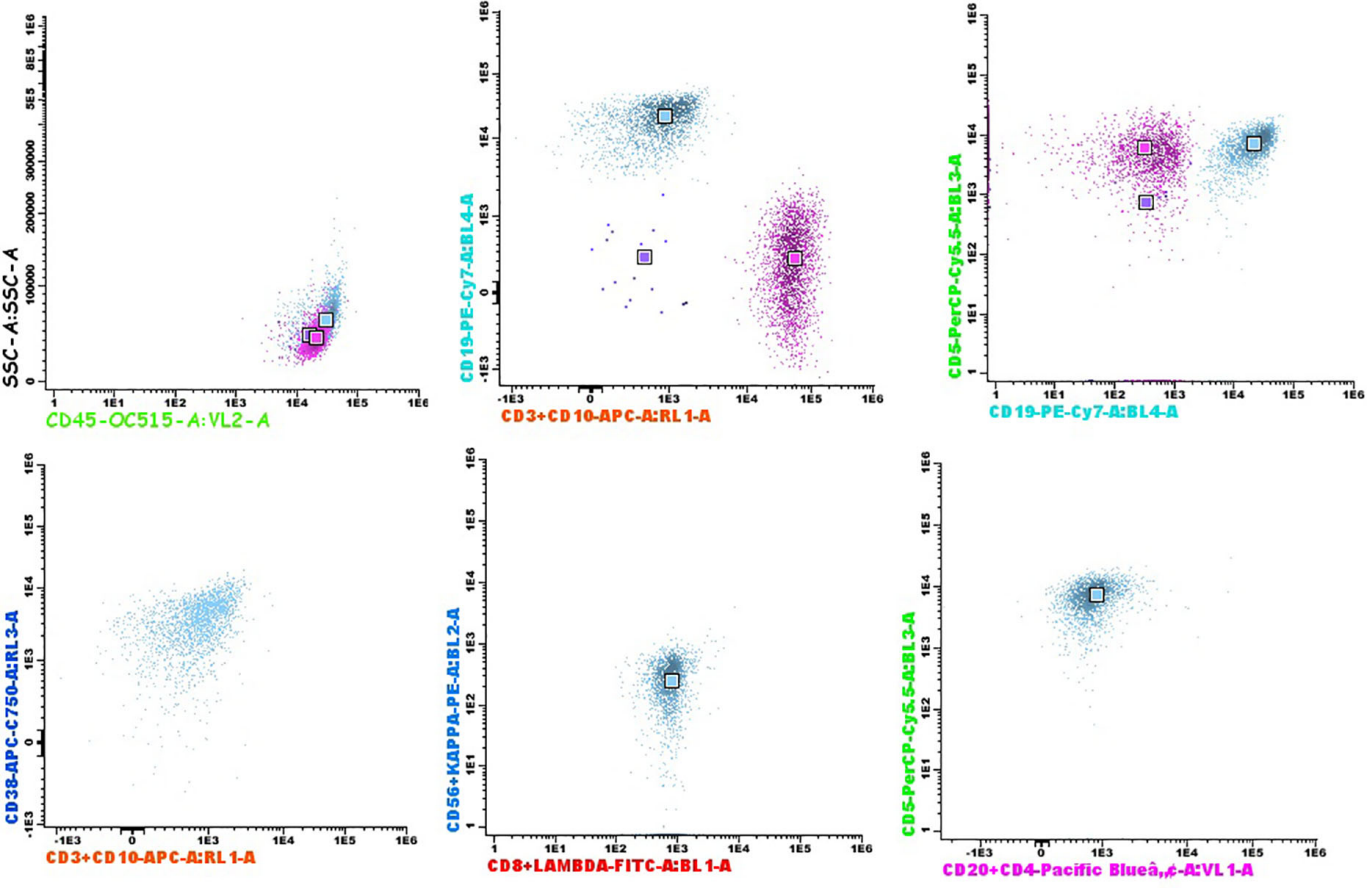
Flow cytometry analysis of vitreous humor. T lymphocytes in pink. Pathological B cells in blue.

Episcleral biopsy was non-diagnostic, prompting diagnostic pars plana vitrectomy in January 2024. The sample was split for cytology and flow cytometric immunophenotyping (FCI). The panel was made with the Lymphoclonal™-12 reagent (BD Cytognos™) which includes: CD3, CD4, CD5, CD8, CD10, CD19, CD20, CD38, CD45, CD56, and κ/λ light chains. Vitreous humor sample showed abundant cellularity (4790 evaluable cells) consisting of lymphocytes, with few monocytes and segmented neutrophils. The lymphocyte population was predominantly T-cell, with an inversion of the CD4/CD8 ratio within the normal range (CD4/CD8 ratio: 0.3). A pathological B lymphoid population of low size and complexity was detected, accounting for 43% of the events (99.6% of B lymphocytes). This population immunophenotype was CD19+, CD20-, CD5+, CD10-, IgG-/d lambda, CD38 ++. The presence of this monoclonal B cell population with κ/λ restriction and an aberrant phenotype consistent with tumor biology was considered diagnostic of lymphomatous infiltration (**Figure 2**).

**Figure 3:**
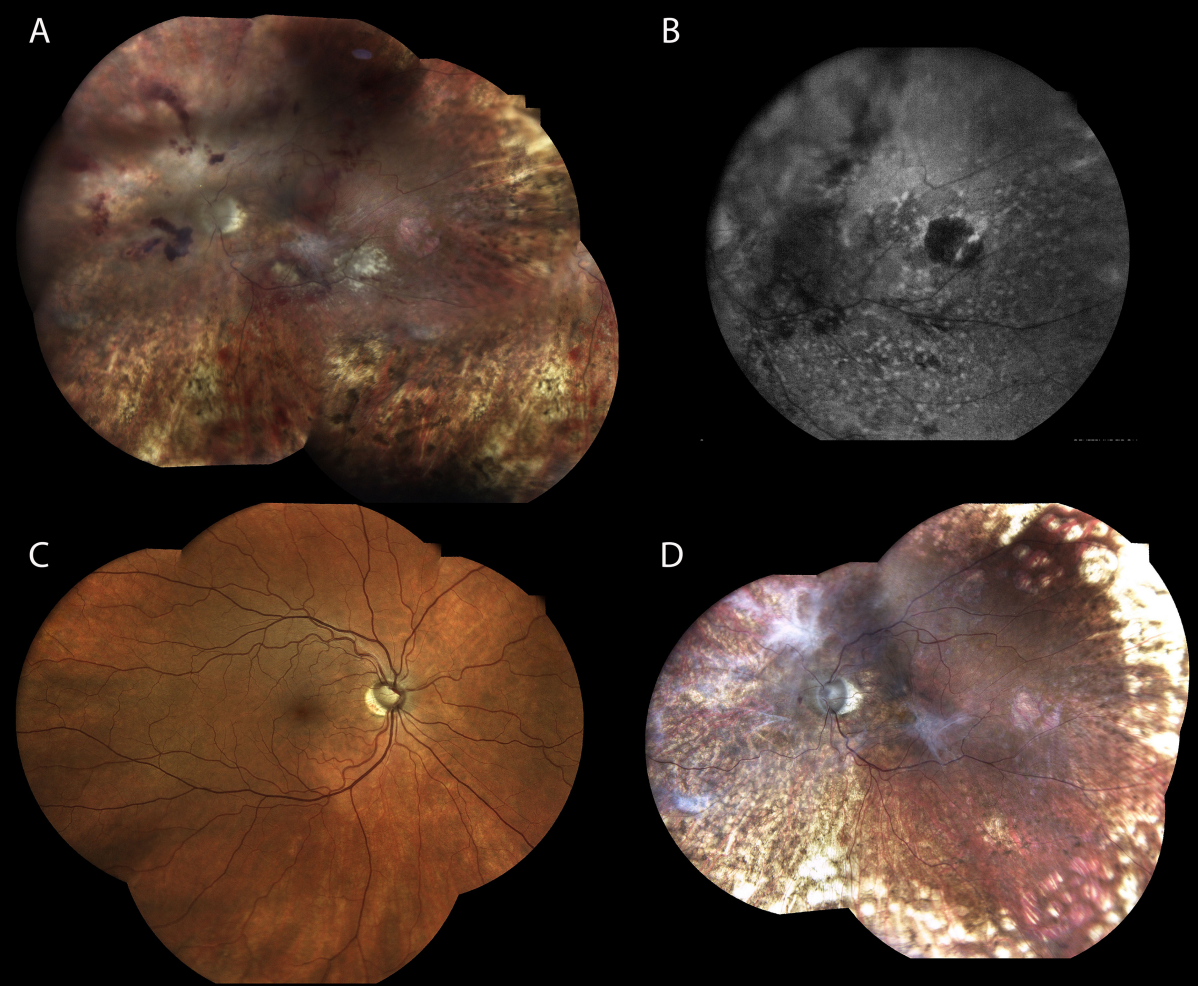
**(A)** Retinography of the LE obtained prior to CAR-T and after diagnostic vitrectomy, followed by four intravitreal methotrexate injections. This image shows the absence of vitreous inflammation; the white spots were retinal laser scars. The biopsy was conducted in an eye affected by media opacity due to vitreous bleeding and exudative retinal detachment, so laser photocoagulation was mandatory. Additionally, there are residual preretinal hemorrhages post-surgery. **(B)** Fundus autofluorescence imaging of the LE demonstrating central retinal atrophy, correlating with the patient’s persistent visual impairment. **(C)** Retinography of the RE showing a normal retinal appearance without inflammatory or infiltrative changes. **(D)** Retinography of the LE after CART therapy showing complete resolution of vitritis, hemorrhages, and retinal detachment.

The patient was accepted for the administration of brexucabtagene autoleucel (Brexucel) by the advanced therapies committee undergoing lymphapheresis in February 2024. Due to the isolated eye relapse, he received only local treatment as bridging therapy. Intravitreal methotrexate (0.4 mg/0.1 mL) was administered once a week during 4 weeks, leading to transient improvement in vitreous clarity (**Figure 3A**). The patient received lymphodepletion (fludarabine and cyclophosphamide), followed by infusion of Brexucel in March 2024.

He experienced grade 1 cytokine release syndrome (CRS) managed with antipyretics and one dose of siltuximab. No neurological toxicity, ocular inflammation or pseudoprogression was observed.

PET-CT showed no signs of systemic progression. Response assessment after CAR-T infusion relied on dedicated ophthalmologic imaging, including fundoscopy and B-scan ultrasonography, both of which documented marked improvement in vitreous opacities and resolution of choroidal thickening (**Figure 3D**). At last follow-up in October 2025, he remained in complete metabolic remission with no evidence of systemic or ocular relapse. Visual function in the left eye was limited to light perception, while the right eye maintained normal visual acuity.

A detailed timeline of the clinical course is provided in **Table 1**.

**Table 1:** Timeline of disease and treatments.

Year	Event/Diagnosis	Treatment/Intervention	Response/Outcome
2018	Stage IV-B MCL (gastric + marrow involvement)	RV-CAP → R-ESHAP → BEAM-ASCT → Rituximab maintenance	Complete remission
2021 Mar	1st relapse (extranodal)	HyperCVAD-R/MTX-AraC-R → Ibrutinib	Complete remission
2022 Jun	2nd relapse (progression on ibrutinib)	Tafasitamab + parsaclisib (clinical trial)	Mixed response → discontinued (hepatitis E)
2023 Nov	Left eye visual loss	Ophthalmologic evaluation → vitrectomy planned	—
2024 Jan-Feb	Vitrectomy confirms intraocular relapse by flow cytometry	Intravitreal methotrexate (bridge)	Partial response (ophthalmological examination)
2024 Mar	CAR-T therapy (Brexucel)	Lymphodepletion + Brexucel infusion	CRS grade 1, no ICANS
2024–2025	Follow-up	PET-CT complete response; persistent visual impairment in LE	Ongoing remissions

## 3 Discussion

Intraocular relapse of MCL is exceptionally rare, with fewer than ten cases reported to date (9, 10). This case underscores both the rarity and the clinical complexity of intraocular MCL relapse.

Our patient exhibited blastoid morphology (Ki-67 70%), indicative of aggressive clinical behavior, and experienced multiple early relapses despite intensive first-line therapy. The combination of blastoid morphology, high proliferation index, and early relapse reflects an inherently aggressive biological profile (11). Notably, CNS prophylaxis was not administered during initial therapy, as there was no evidence of ocular or neurological involvement at that time. Nonetheless, the role of prophylaxis in high-risk blastoid MCL remains controversial, particularly in light of emerging evidence of occult CNS dissemination. Current ESMO and BSH guidelines report CNS relapse in MCL as uncommon (~3–5%) and do not recommend routine prophylaxis unless neurological symptoms or high-risk features are present (12, 13). Consistent with these recommendations, CNS-directed therapy was deemed unnecessary in this case (14).

Differentiating lymphoma from inflammatory or infectious etiologies requires a high degree of clinical suspicion and a multimodal diagnostic approach. The current gold standard combines cytology, immunohistochemistry, flow cytometry, and molecular studies. Flow cytometry enhances sensitivity compared to cytology alone, while IL-10 measurement and molecular assays further improve diagnostic accuracy (15–17).

The marked retinal and choroidal atrophy likely reflects a combination of prior exudative retinal detachment, ischemic damage secondary to vasculitis/periphlebitis, surgical intervention (vitrectomy and laser photocoagulation), and local methotrexate toxicity, all of which are recognized causes of structural retinal damage in vitreoretinal lymphoma.

In this case, vitreous FCI was decisive, identifying a monoclonal B-cell population consistent with the patient’s prior MCL phenotype (CD19^+^, CD5^+^, CD20^-^, λ restricted) and thereby confirming ocular relapse despite nondiagnostic cytology. Although FCI is dependent on sample quality, it significantly increases diagnostic yield relative to cytology alone.

Evidence from the Lymphome Oculo-Cérébral (LOC) network suggests that IL-10 clearance post–CAR-T correlates with sustained remission (18).

Importantly, the pre–CAR-T PET-CT did not detect the intraocular relapse, consistent with the known limitations of PET imaging in small-volume ocular disease (19).

Most available data on CAR-T therapy in intraocular lymphoma come from patients with DLBCL or PCNSL. Notably, the LOC network’s seminal report on CAR-T efficacy in vitreoretinal lymphoma included only DLBCL cases, with no representation of MCL (20). This case demonstrates that CAR-T cells can achieve sustained remission even in an immune-privileged site, extending the evidence beyond the previously reported DLBCL/PCNSL cases (10, 21).

Nevertheless, important limitations remain. This is a single-patient report, and the contribution of bridging intravitreal methotrexate to local disease control cannot be fully excluded. Moreover, standardized ocular response criteria, CAR-T pharmacokinetics in vitreous/aqueous compartments, and long-term safety profiles remain undefined, underscoring the need for collaborative registries and prospective studies (6, 22). Although intravitreal methotrexate could have contributed to initial disease control, the durability of response (>18 months) strongly supports CAR-T therapy as the primary driver of remission (23). Real-world data confirm similar efficacy with manageable CRS and neurotoxicity (24).

In intraocular lymphoma, additional challenges arise. In DLBCL, the LOC network reported seven patients with vitreoretinal lymphoma treated with anti-CD19 CAR-T cells, achieving an ocular response rate of 83% and two-year overall survival of 83%, with CAR-T cells detected in the aqueous humor, confirming effective biodistribution (22, 25).

While prior reports have suggested CAR-T activity in ocular involvement by DLBCL or PCNSL (26), this represents one of the first documented cases demonstrating durable intraocular remission in MCL.

In summary, this case highlights the importance of early recognition and precise diagnostic confirmation, particularly the value of flow cytometry, in intraocular MCL relapse. It demonstrates that CAR-T therapy can achieve durable remission in immune-privileged compartments and underscores the need for multidisciplinary collaboration. These findings support further studies to standardize ocular endpoints, monitor CAR-T persistence in ocular fluids, and evaluate long-term visual and systemic outcomes.

## Data Availability

The original contributions presented in the study are included in the article/Supplementary Material. Further inquiries can be directed to the corresponding author.
